# Migrants, refugees and displaced populations: a missed opportunity for equity at the 2025 UN High-Level Meeting on NCDs and mental health

**DOI:** 10.1016/j.lanepe.2025.101558

**Published:** 2025-12-09

**Authors:** Santino Severoni

**Affiliations:** WHO Special Initiative on Health and Migration, World Health Organization, Geneva, 1202, Switzerland

The United Nations High-Level Meeting (HLM) on Noncommunicable Diseases (NCDs) and Mental Health was held on 25 September 2025. Unlike previous meetings, this HLM concluded without the adoption of a Political Declaration—a consensus document that outlines global commitments and strategies to address NCDs and mental health. Instead, the draft Declaration will proceed to the United Nations General Assembly for further consideration and possible revision. This unusual postponement at the HLM stage highlights both the contentious nature of current negotiations and the urgency of ensuring that any final agreement prioritises equity and inclusivity in global health responses. Yet even in its current form, the draft Declaration exposes a profound gap: the absence of explicit commitments for migrants, refugees, and displaced populations.

This omission turns a moment of promise into a missed opportunity—weakening equity, undermining global progress on Universal Health Coverage (UHC) and the Sustainable Development Goals (SDGs), and contradicting our collective pledge to leave no one behind.

## The case for inclusion

The magnitude of migration and displacement today makes their exclusion untenable. More than 1 billion people are migrants or displaced, including 304 million international migrants (2024) and 123.2 million forcibly displaced by conflict and persecution (2024).^1^^,^^2^ This is not a marginal population—it represents nearly one in eight people worldwide. Refugees and migrants are disproportionately exposed to the social and structural determinants of NCDs—poverty, poor nutrition, chronic stress, and unsafe living and working conditions—placing them at higher risk of hypertension, diabetes, and depression than host populations.^3^^,^^4^

Beyond elevated risk, major gaps in continuity of care persist. Fragmented health systems and weak cross-border coordination disrupt treatment for lifelong conditions such as diabetes, cancer, and cardiovascular disease.^3^ Interruptions in treatment cost lives and increase health system costs through avoidable complications.^3^ Moreover, climate change further multiplies these risks. By 2050, climate-related displacement may exceed 200 million people, many of whom will face aggravated cardiovascular, respiratory, and mental health challenges.^5^

## The economics of action versus inaction

The economic rationale for inclusion is equally important. NCDs already account for 74% of all deaths globally and are projected to cost the world economy over US$ 47 trillion by 2030 in lost productivity and health care expenditure if current trends continue.^6^ Mental disorders are expected to cost the global economy US$ 16.3 trillion between 2011 and 2030.^4^ Yet action is both feasible and affordable. Including migrants and refugees is cost-saving and strengthens health system efficiency. WHO estimates that a package of “best buy” NCD interventions—including screening, treatment of hypertension, diabetes, and depression—costs less than US$ 1 per person per year in low- and middle-income countries.^7^

Excluding migrants and refugees results in delayed diagnosis, emergency-only care, and higher system costs. Studies in Europe show that providing preventive and primary care to migrants is significantly more cost-effective than emergency-only access, reducing per-patient costs by up to 50% over time.^8^ Mental health investments show a similarly high return: every US$ 1 invested in scaling up treatment for common mental health conditions yields a return of US$ 4 in improved health and productivity.^9^ Failing to extend these services to migrants and refugees wastes potential economic and social gains.

Inclusion is therefore not only a matter of rights—it is sound economics. The cost of inaction is far greater than the cost of action.

## Political and normative momentum

Migrants and refugees are already recognized in the Political Declarations on Universal Health Coverage (2019, 2023), Pandemic Preparedness (2023), Tuberculosis (2023), and Antimicrobial Resistance (2024). The Rabat Declaration on Migration Health and Rights (2023) reaffirmed Member States’ commitment to equitable access to health for migrants, including NCD prevention and mental health. To omit them now would be a step backwards, undermining the coherence of UN commitments.

Box 1 **The way forward**

The adoption of today's Political Declaration opens space for building more inclusive approaches to NCDs and mental health. Ensuring that migrants, refugees, and displaced populations are not left behind will depend on how the commitments are implemented in practice. Key opportunities include.

• Embedding inclusion in national strategies so that migrants and refugees are visible in monitoring, reporting, and accountability processes.
• Enhancing continuity of care across borders, ensuring access to lifelong treatment and mental health support through interoperable health information systems, portable health records, and regional referral networks.
• Fostering whole-of-government approaches that link health with education, labor, housing, and social protection to address structural determinants of inequity.
• Exploring sustainable financing pathways by leveraging international partnerships and innovative funding models.^10^
• Encouraging migrant and refugee participation in shaping policies and programmes that affect their health and well-being.

## A pivotal opportunity

Migrants and refugees strengthen societies economically, socially, and culturally. Their health is an investment in resilience and inclusive growth. Excluding them risks perpetuating inequities, driving up health system costs, and slowing global progress on NCDs, mental health, UHC, and the SDGs.

As the draft Political Declaration now moves to the General Assembly for final consideration, there is still a pivotal opportunity to strengthen its equity lens. Explicit inclusion of migrants, refugees, and displaced populations will not only reinforce global commitments but also create a powerful domino effect—shaping national and local policies, guiding resource allocation, and influencing health system reforms. The credibility and impact of the Declaration will depend on how inclusively it is finalized and implemented. Ensuring that migrants, refugees, and displaced populations are at the center of this process can transform the Declaration from a missed opportunity into a driver of equity, resilience, and shared progress.

## Declaration of interests

SS is the Head of WHO Special Initiative on Health and Migration, WHO. The author alone is responsible for the views expressed in this Viewpoint, which do not necessarily represent the decisions, policy, or views of WHO.
